# The Clinical Interpretation of Research

**DOI:** 10.1371/journal.pmed.0020395

**Published:** 2005-11-29

**Authors:** Stephen G Pauker

**Affiliations:** **1**Tufts-New England Medical CenterBoston, MassachusettsUnited States of America

John P. A. Ioannidis emphasizes the central role of prior probabilities [1]. His conclusion rests on the presumed low probability that a hypothesis was true before the study.

Unfortunately, his formulation relates the post-study probability that the study's conclusion is true to the pre-study odds. The results might have been clearer had he also plotted the relation of odds to probability, a curvilinear relationship, assuming the study carried no information. Further, the various graphs are right-truncated at pre-study odds, *R*, of 1.0 (a probability of 0.5), although his examples go as high as *R* = 2.0. A positive study must, by definition, increase the likelihood that the hypothesis is true. It might have been clearer had Ioannidis chosen to relate odds to odds or probability to probability; in both cases, a neutral study would produce a straight line along a 45-degree diagonal.

The pre-study to post-study relation can more simply be expressed using the odds-likelihood form of Bayes rule—i.e., the post-study odds equal the pre-study odds multiplied times the likelihood ratio (LR) of the study. Then, the equations for positive predictive value (PPV) become the simple product of *R* × LR. For a single unbiased study, LR = (1 − β)/α. When incorporating study bias, *u*, as defined by Ioannidis, LR = (1 − β[1 − *u*])/(α[1 − *u*] + *u*). For a typical study with α = 0.05 and β = 0.2 (i.e., with a power of 0.8), LR = 16. When *R* is less than 1:16 (a probability of 0.0588), the post-study odds will be less than one—i.e., the study's hypothesis will be more likely false than true.

For non-Bayesians, statistical significance testing presumes uninformative prior probability—i.e., *R* = 1. Then, LR would merely need to exceed one for the study's conclusions to be more likely true than false. At the common significance levels (α) of 0.05 and 0.01, the requisite study powers would merely need to exceed 0.05 and 0.01 respectively, corresponding to maximum type II error rates (β) of 0.95 and 0.99. Such lax requirements would almost always be met for a published study. Hence, the common belief that the vast majority of studies have valid conclusions would be correct if we can assume that the pre-study odds are truly uninformative. However, as Ioannidis suggests, this is unlikely to be the case.

Two more corollaries might be added. The higher the pre-study odds that the study's hypothesis is true, the lower the requisite power (study size and effect size) required to make the study's findings more likely true than false. When studies are published, the investigator should estimate the pre-study odds and report the LR implied by the observed effect.

From the perspective of an epidemiologist or a statistician, the relevant question is whether the study's hypothesis is true—i.e., is the probability of the hypothesis greater than 0.5? For clinicians and their patients, the relevant question is whether a particular strategy should be followed in an individual patient or a subset of similar patients. That decision (or recommendation to the patient) will depend on the pre-study likelihood of benefit in that patient and on the relative magnitude of benefits and risks of that strategy, if the diagnosis in that patient is uncertain. For many such decisions, the “more likely true than false” criterion may not be the best decision rule. For serious diseases and treatments of only modest risk, post-study probabilities of considerably less than 0.5 may be sufficient to justify treatment [2].

Ioannidis's provocative Essay is a timely call for careful consideration of published studies. The odds-likelihood formulation suggested herein may be helpful in providing a more intuitive model. Clinicians now need to take it to the next step.
